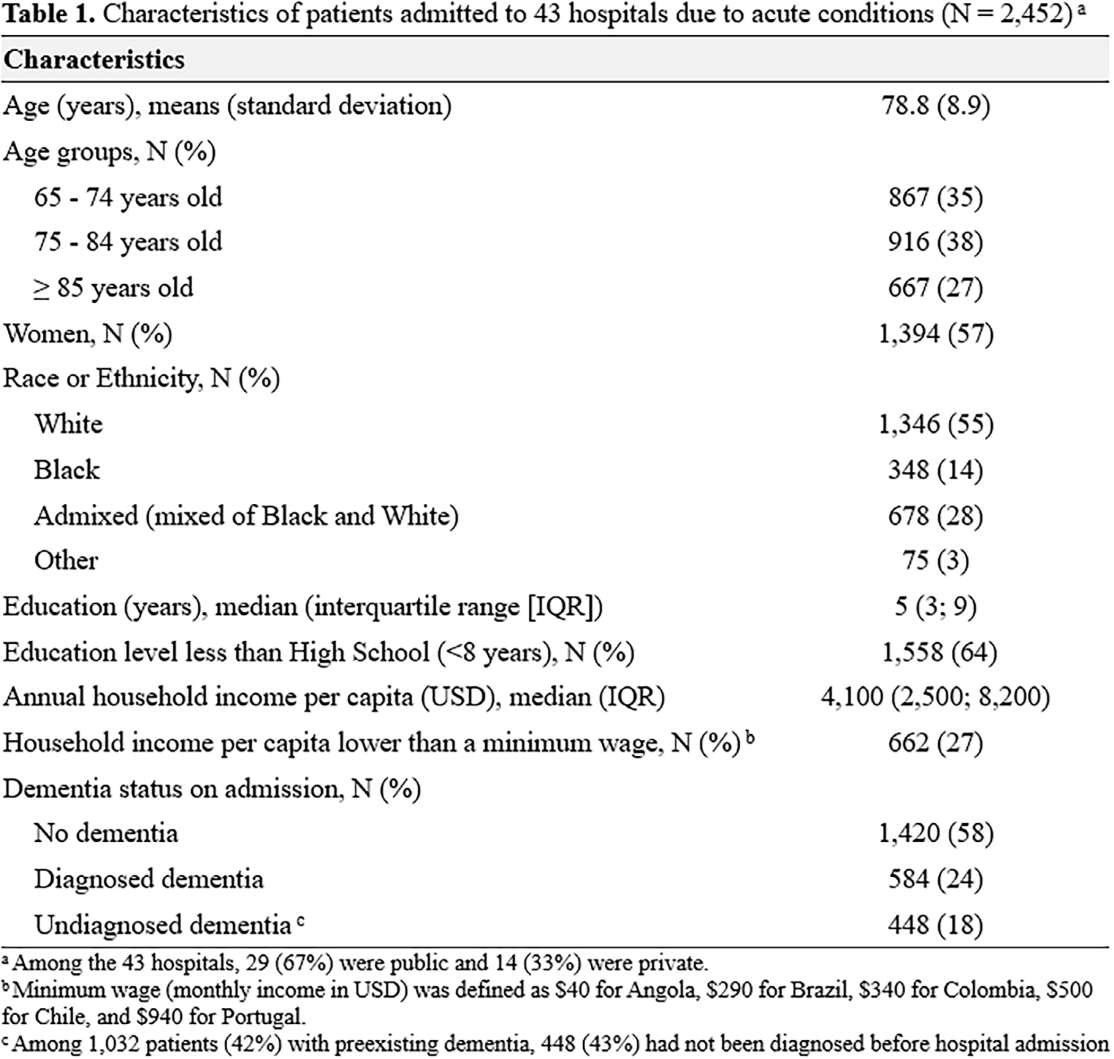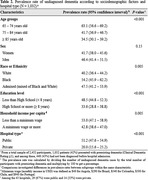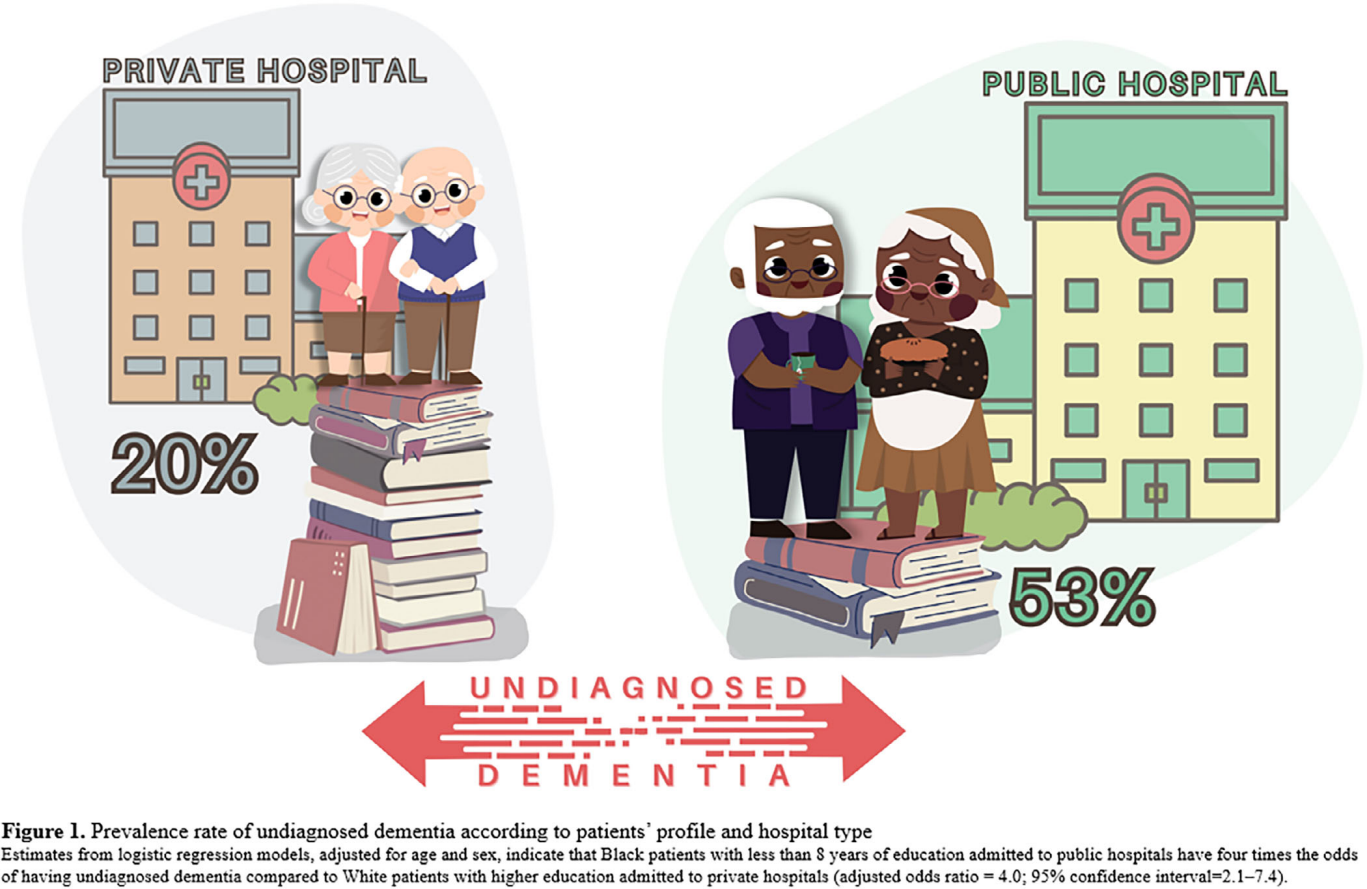# Prevalence of undiagnosed dementia among older hospitalized patients: variation by social and demographic factors

**DOI:** 10.1002/alz.088225

**Published:** 2025-01-09

**Authors:** Márlon Juliano Romero Aliberti, Thiago J Avelino‐Silva, Kenneth E Covinsky, Claudia Kimie Suemoto, CHANGE Study Group

**Affiliations:** ^1^ University of São Paulo Medical School, São Paulo, São Paulo Brazil; ^2^ Hospital Sírio‐Libanês, São Paulo, São Paulo Brazil; ^3^ University of Sao Paulo Medical School, Sao Paulo, Sao Paulo Brazil; ^4^ University of California San Francisco, San Francisco, CA USA

## Abstract

**Background:**

The profound impact of dementia on acute care, compounded by frequent underdiagnosis, is a significant challenge, especially among certain ethnic and minority groups, and remains largely unexplored in hospital settings. We used data from a multicentric study comprising 43 public and private hospitals in five countries to estimate the prevalence of undiagnosed dementia across different sociodemographic measures.

**Method:**

The CHANGE (Creating a Hospital Assessment Network in Geriatrics) Study, an ongoing cohort designed to identify age‐related conditions like dementia, included patients aged ≥65 years admitted to 43 acute hospitals throughout Brazil and four other countries: Angola, Chile, Colombia, and Portugal. Upon admission, participants underwent a comprehensive geriatric assessment. To determine a previous dementia diagnosis, we consulted close family members and reviewed medical records. Trained investigators also administered the Clinical Dementia Rating (CDR) to family informants, referencing the patients’ cognitive status three months before admission to avoid the influence of acute cognitive impairment. Undiagnosed dementia was defined for participants with no clinical history of dementia and a CDR score ≥1. We estimated the prevalence of undiagnosed dementia across different sociodemographic measures and hospital types.

**Result:**

In a sample of 2,452 participants (mean age = 79±9 years; women = 57%; Black or admixed = 42%), 1,032 (42%) presented with preexisting dementia (CDR ≥1), and among these, 448 (43%) had undiagnosed dementia (Table 1). Although no significant difference was found based on sex, undiagnosed dementia was associated with younger age, Black or admixed race/ethnicity, lower education levels, and lower income (Table 2). Moreover, the rate of undiagnosed dementia was more than double in public hospitals (47%) compared to private hospitals (22%), with a p‐value<0.001. Notably, Black patients with less than 8 years of education in public hospitals were significantly more likely to have undiagnosed dementia compared to White patients with higher education admitted to private hospitals, with undiagnosed dementia rates of 53% versus 20%, respectively (p‐value<0.001) (Figure 1).

**Conclusion:**

Four out of ten older patients with preexisting dementia had not been diagnosed prior to hospital admission, underscoring the urgent need for practical diagnostic approaches to improve care in acute settings, particularly for ethnic and socioeconomic minorities in public hospitals.